# Impact of Harvest Delay and Barley Variety on Grain Nutritional Composition and Mycotoxin Contamination

**DOI:** 10.3390/jof10110738

**Published:** 2024-10-24

**Authors:** Eimantas Venslovas, Yuliia Kochiieru, Sigita Janavičienė, Lauksmė Merkevičiūtė-Venslovė, Mohammad Almogdad, Vadims Bartkevics, Zane Bērziņa, Romans Pavlenko

**Affiliations:** 1 Lithuanian Research Centre for Agriculture and Forestry, Kėdainiai Distr., LT-58344 Akademija, Lithuania; yuliia.kochiieru@lammc.lt (Y.K.); sigita.janaviciene@lammc.lt (S.J.); mohammad.almogdad@lammc.lt (M.A.); 2 Institute of Food Safety, Animal Health and Environment “BIOR”, LV-1076 Riga, Latvia; vadims.bartkevics@bior.lv (V.B.); zane.berzina@bior.lv (Z.B.); romans.pavlenko@bior.lv (R.P.)

**Keywords:** agricultural practices, biochemical analysis, crop maturity, fungal toxins, meteorological conditions, nutritional composition

## Abstract

This study investigated the effects of delayed harvesting, varying meteorological conditions, and barley variety on *Fusarium* spp. infection rates, nutritional composition, and mycotoxin contamination in barley grains. Field experiments were conducted from 2020 to 2022 and involved two barley varieties: ‘Laureate’ for malting and ‘Luokė’ for feed. The results indicated that the dominant *Fusarium* species isolated were *F. avenaceum*, *F. culmorum*, *F. poae*, *F. sporotrichioides*, *F. tricinctum*, and *F. equiseti*. These tended to increase in number with delayed harvest times and were more prevalent during harvest periods of higher precipitation (*p* < 0.05). Malting barley had higher starch and lower protein content compared to feed barley (*p* < 0.05). Delayed harvesting generally increased dry matter, crude fat, and crude ash contents while decreasing crude protein, zinc, and iron contents (*p* < 0.05). Mycotoxin analysis revealed significant differences under specific weather conditions. HT-2 toxin levels were higher under slightly warmer and wetter conditions during flowering, with harvest conditions similar to the long-term average. Zearalenone levels increased with dry, warm growing seasons followed by rainy harvests. Nivalenol and enniatin levels increased with rainy growing seasons and dry, warm harvests. Deoxynivalenol concentrations did not reach the limit of quantification throughout the study. No consistent trend was observed for higher contamination in any specific barley variety (*p* > 0.05). The strongest correlations between mycotoxins and nutritional value indicators were observed with less-studied mycotoxins, such as nivalenol and enniatins, which exhibited negative correlations with crude protein (*p* < 0.01), crude fat (*p* < 0.05), and zinc (*p* < 0.01), and positive correlations with crude ash (*p* < 0.05) and phosphorus (*p* < 0.01).

## 1. Introduction

Barley (*Hordeum vulgare* L.) is a cereal grain of considerable agronomic and economic significance due to its adaptability to diverse climates and soil conditions [1]. It serves as a crucial protein and energy source for livestock such as beef cattle, pigs, and poultry, and is utilized in the food industry for malt production, as well as culinary applications including bread, stews, soups, and health-oriented products [2,3].

The chemical composition of barley grains varies significantly among different varieties, influencing their nutritional digestibility [4]. Barley grain is rich in dietary fiber and antioxidants, such as phenolic compounds, contributing substantially to the crop’s nutritional value. It contains significant amounts of unsaturated fatty acids and carotenoids, which offer various health benefits [5]. Typically, barley grain contains approximately 10.0–12.8% protein, around 60% starch, 1.9–2.6% fat, 2.0–2.5% ash, and 4.0–5.7% fiber [6,7]. These levels are influenced by factors such as variety, genotype, farming practices, and environmental conditions [5].

Cereal crops, including barley, are susceptible to fungal infections caused by species such as *Aspergillus*, *Monascus*, and *Fusarium*. These fungi, as a group, produce mycotoxins, including aflatoxins (AFLs) (B1, B2, G1, G2), deoxynivalenol (DON), zearalenone (ZEA), T-2 toxin, and HT-2 toxin, which pose significant global health risks [8,9,10]. The consumption of grains heavily contaminated with mycotoxins can lead to chronic and acute diseases or even death in humans and animals [11]. Moreover, various mycotoxins are known for their carcinogenic, mutagenic, teratogenic, hepatotoxic, and estrogenic effects [12]. Given that heavily contaminated grains are unsuitable for consumption and the detoxification or decontamination of such grains remains a global challenge, the economic implications are substantial [13]. Therefore, the European Commission has established maximum allowed and recommended concentrations for specific mycotoxins in food and feed [14,15,16,17].

Implementing good agricultural and storage practices is crucial for mitigating mold growth and mycotoxin contamination [18]. Tillage practices have been shown to influence mycotoxin levels; for instance, research in Lithuania indicated that no-till farming resulted in DON and ZEA concentrations in triticale being at least three times higher than those in conventional farming systems [19]. The timing of harvest and preventing mechanical damage during the growing season are also critical considerations [20]. Proper plant care is critical to protect against pests, while ensuring optimal storage conditions, including moisture and temperature control, provides protection from storage molds, insects, and rodents [21]. The continuous monitoring and management of conditions that can lead to increased mycotoxin concentrations are crucial to prevent grain contamination [22].

Genetics also play a significant role in the susceptibility to fungal infections and mycotoxin production. For example, high expression of the nepenthesin-1 gene in barley grain is associated with reduced *Fusarium* head blight incidence [23]. German researchers have identified variations in *Fusarium* spp. infection rates among spring barley genotypes, with higher infection rates correlating with increased DON, 3-acetyl-deoxynivalenol (3-ADON), and 15-acetyl-deoxynivalenol (15-ADON), though no significant differences were observed for ZEA and nivalenol (NIV) concentrations among the tested genotypes [24]. The aim of this study was to investigate the impact of delayed harvesting, varying meteorological conditions, and barley variety on *Fusarium* spp. infection rates, nutritional composition, and mycotoxin contamination in barley grain.

## 2. Materials and Methods

### 2.1. Field Experiments

Field experiments were conducted at the experimental fields of the Plant Pathology and Protection Department of the Lithuanian Research Centre for Agriculture and Forestry from 2020 to 2022. A total of 72 spring barley grain samples were collected. Two different spring barley varieties were selected for the study: ‘Laureate,’ known for its high quality and use in malting, and ‘Luokė,’ valued for its robustness and use as feed. These varieties were chosen because they are among the most commonly grown in Lithuania, representing both key agricultural markets in the region. ‘Luokė’ has resistance to spot blotch, *Septoria* leaf blotch, and scald, while ‘Laureate’ has resistance to blotch, powdery mildew, scald, and *Ramularia* leaf spot. Flowering dates were between 23 June and 2 July. Harvesting took place in three stages: at hard maturity (1st, BBCH 89—the grain is hard and difficult to press with a fingernail), 10 (±2) days after the first harvest (2nd), and 20 (±2) days after the first harvest (3rd) (Table 1). A randomized complete block design was used to set up the trial. Plots of two spring barley varieties were sown for the three planned harvest times; each plot measured 2.5 m in width and 10 m in length, with four replications. The seeding rate was about 200 kg ha^−1^. The soil at the local site is classified as Cambisol—specifically, a drained, Endocalcaric, Endogleyic loam. It has a neutral pH and is moderately rich in humus. Fertilization and the use of plant protection products were carried out in accordance with the cultivation recommendations for barley. The fields were initially fertilized before sowing with granular NPK 6-18-34 at a rate of 400 kg ha^−1^. One week after sowing, granular ammonium nitrate was applied at 150 kg ha^−1^. Herbicides were then sprayed, consisting of 55 g ha^−1^ tritosulfuron (714 g kg^−1^) and florasulam (54 g kg^−1^), mixed with 1L ha^−1^ of MCPA (500 g L^−1^) and 0.5 L ha^−1^ of surfactants (99.6% alkylphenol ethoxylates). Growth regulators (trinexapac-ethyl 250 g L^−1^) at 0.4 L ha^−1^ were sprayed one week later. In mid-June, a second application of granular ammonium nitrate at 150 kg ha^−1^ was applied. Insecticides were sprayed as follows: in 2020, thiacloprid (100 g L^−1^) and deltamethrin (10 g L^−1^) at 0.75 L ha^−1^; in 2021, beta-cyfluthrin (25 g L^−1^) at 0.3 l ha^−1^; and in 2022, cyhalothrin (50 g kg^−1^) at 1.2 L ha^−1^. By the end of June, fungicides, were sprayed. In 2020, protioconazole (150 g L^−1^) and benzovindiflupyr (75 g L^−1^) were sprayed at 1 L ha^−1^. In 2021 and 2022, protioconazole (160 g L^−1^) and spiroxamine (300 g L^−1^) were sprayed at 1 L ha^−1^. Barley grain was harvested using combines. Samples were ground using an Ultra Centrifugal Mill ZM 200 (Retsch, Haan, Germany) and stored at −20 °C until laboratory analysis.

### 2.2. Meteorological Conditions

**Figure 1:**
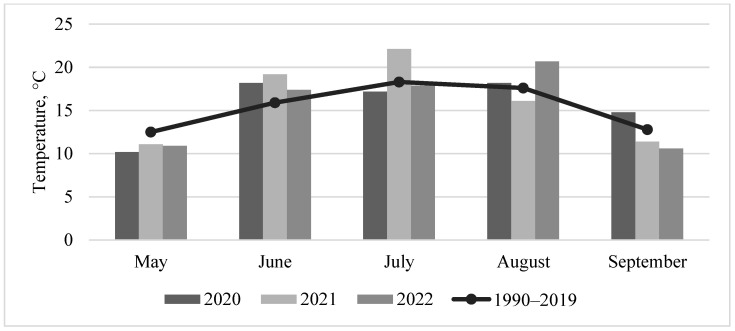
Average monthly air temperatures during the barley vegetation seasons from 2020 to 2022 compared to the long-term average monthly air temperatures (1990–2019).

**Figure 2:**
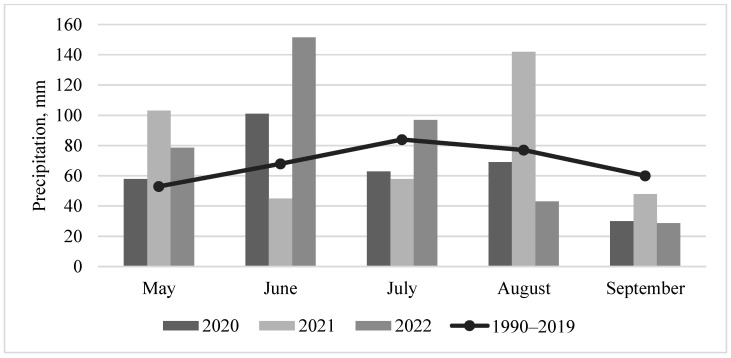
Total monthly precipitation during the barley vegetation seasons from 2020 to 2022 compared to the long-term average total monthly precipitation (1990–2019).

Based on the meteorological observations from the local station in Dotnuva (55°23′49.0″ N 23°51′55.0″ E, Kėdainiai district), the weather patterns in 2020, 2021, and 2022 exhibited distinct characteristics. During the beginning of the barley growing season in 2020, temperatures were below the long-term average in May (Figure 1). June and September experienced hotter-than-normal temperatures, while the other months remained close to the long-term average. Additionally, June experienced higher-than-average precipitation, whereas July, August, and September had precipitation levels below the long-term average (Figure 2). The second year of the study (2021) was exceptional. Temperatures at the beginning of the barley growing season were slightly below the long-term average, but above average in June and July. Rainfall was greater than normal for May and August, but below the long-term average in June and July in 2021. Consequently, dry and warm weather predominated during barley flowering and the late grain filling period. During the barley harvest or just prior to it, conditions were wetter and slightly cooler compared to 2020 and 2022. At the beginning of the barley growing season in May 2022, temperatures were below the long-term average temperature, as observed in every year of the study. In June and July of 2022, temperatures were close to or slightly above the long-term average, accompanied by higher-than-normal rainfall from May to July. During the 2022 barley harvest in August and September, rainfall decreased, and temperatures were higher or slightly below the long-term average.

### 2.3. Nutritional Value Analyses

Dry matter (DM) content was determined by drying at 105 °C to a constant weight. Starch content was measured using the polarimetric method according to LST EN ISO 10520:2000 [25]. Crude fiber (CF) was analyzed gravimetrically according to LST EN ISO 16472:2006 [26]. Crude fat was determined by modified Soxhlet extraction method LST EN ISO 11085:2016 [27]. The crude ash (CA) content in the samples was determined using the gravimetric method LST EN ISO 2171:2023 [28]. The samples were incinerated at 550 °C, and the remaining residue was weighed to calculate the ash content. Crude protein (CP) content was determined by the Kjeldahl method according to LST EN ISO 5983-1:2005 [29]. The contents of calcium, zinc, iron, and magnesium were measured using a PerkinElmer AAnalyst 200 (Shelton, CT, USA) atomic absorption spectrometer from SpectraLab (Markham, ON, Canada) [30]. Phosphorus content was determined spectrophotometrically [31]. In the 3-year experiment, 24 barley grain samples were collected each year, resulting in a total of 72 samples. All chemical analyses were performed with two replications, with a total of 144 samples for each analysis.

### 2.4. Fusarium spp. Rating

The agar plate method was used to assess internal grain infection. Grain surfaces were sterilized in a 1% NaOCl solution for 3 min, after which 100 grains per sample were plated in Petri dishes on potato dextrose agar (PDA) amended with 1.6 mL L^−1^ Triton X-100 and 3 mL L^−1^ of 20% citric acid monohydrate. The plates were incubated in the dark at 26 ± 2 °C for 7 days [32]. The *Fusarium* colonies that developed were isolated and purified by sub-culturing onto fresh PDA plates to obtain pure stains. Macroscopic identification was initially based on colony characteristics such as color, texture, and growth pattern. Microscopic examination was performed using a Nikon Eclipse E200 optical microscope (Tokyo, Japan), focusing on conidial features including shape, size, septation, and other morphological structures, and the percentage of contaminated grains was calculated. The colonies were identified using the guides by Nelson et al. [33] and Leslie and Summerell [34].

### 2.5. Mycotoxin Analyses

The concentrations of DON, T-2, HT-2, ZEA, moniliformin (MON), NIV, and enniatins (ENNs) (B, B1, A, A1) were determined at the BIOR Institute of Latvia using an UltiMate 3000 HPLC system coupled with a Thermo TSQ Quantiva triple quadrupole mass spectrometer (Waltham, MA, USA). For the determination of mycotoxins, we used previously optimized and approved system settings and sample preparation methods, including QuEChERS extraction [35]. Mycotoxin standards were obtained from Sigma Aldrich (Steinheim, Germany), and barley reference materials were procured from Trilogy (Washington, MO, USA). Five-point calibration curves were constructed from standard solutions by gradual dilution. Standard solutions for DON, MON, and ENNs (B, B1, A, A1) were prepared at concentrations of 10, 50, 100, 250, and 500 µg kg^−1^; for ZEA, at 100, 250, 500, 750, and 1000 µg kg^−1^; for AFLs, at 0,6, 1,7, 5, 15, and 45 µg kg^−1^; for T-2 and HT-2, at 10, 20, 50, 100, and 200 µg kg^−1^; and for NIV, at 10, 25, 50, 100, and 200 µg kg^−1^. The limits of detection (LOD) and limits of quantification (LOQ) were calculated in µg kg^−1^ using calibration curves: DON (LOD = 48, LOQ = 144), T-2 (LOD = 9, LOQ = 27), HT-2 (LOD = 10, LOQ = 31), ZEA (LOD = 10, LOQ = 30), AFLs (LOD = 0.5, LOQ = 1.5), MON (LOD = 0.6, LOQ = 1.9), NIV (LOD = 3.9, LOQ = 11.7), ENN B (LOD = 3.5, LOQ = 10.5), B1 (LOD = 3.0, LOQ = 9), A (LOD = 1.9, LOQ = 5.7), and A1 (LOD = 1.7, LOQ = 5.1). The coefficient of determination (r^2^) for the calibration curve was at least 0.999. The recoveries of certified reference materials ranged from 81% to 110%. The precision of the method, expressed as relative standard deviation, was less than 11%.

### 2.6. Statistical Analysis

Statistical analyses were conducted using IBM SPSS Statistics (IBM Inc., Armonk, NY, USA) and Microsoft Excel (Microsoft, Redmond, WA, USA). The experimental design used was a randomized complete block design (RCBD). Data from different years were analyzed separately due to the variability in mycotoxin presence, which was influenced by differing weather conditions each year. This year-to-year variability necessitated separate analyses, as combining the data would obscure significant results related to specific harvest times or conditions. Tests for normality and homogeneity of variance were performed to ensure the data met the assumptions for parametric testing. The Shapiro–Wilk test assessed normality, while Levene’s test verified the homogeneity of variance across groups. A two-way ANOVA was employed to assess the interaction effects between barley varieties (malt vs. feed) and harvest dates (hard maturity, +9–10 days later, or 18–22 days later). However, no significant interactions were found, justifying the use of one-way ANOVA for subsequent analysis. Duncan’s post hoc test was applied following the ANOVA to determine significant differences in *Fusarium* species infection rate, nutritional value, and mycotoxin concentrations across different harvest times. The F-test within the ANOVA framework was used to assess the main effects of variety and harvest date, with mean separation tests (Duncan’s post hoc) conducted if the overall F-test was significant. Data are presented as mean ± standard deviation. For values below the LOQ, LOQ/2 was used as a substitute to include these values in statistical analyses. Different lowercase or uppercase letters indicate statistically significant differences among means (*p* < 0.05). Pearson’s correlation coefficient was used to evaluate the relationships between nutritional values and mycotoxin concentrations.

## 3. Results

**Figure 3:**
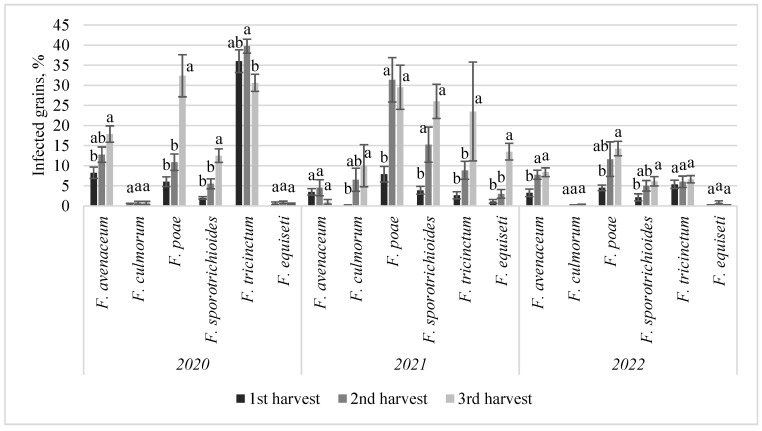
Infection of barley grains by specific *Fusarium* spp. across different harvest times from 2020 to 2022. Note. Different lowercase letters indicate statistically significant differences for each particular *Fusarium* spp. infection between harvests within the same year.

The highest *Fusarium* spp. infection intensity in the grains of both cultivars was observed in 2020 and 2021, both of which had more precipitation during the harvest period, despite experiencing very different weather conditions during flowering. However, no significant difference in infection intensity was detected between the cultivars. The predominant species observed in the grain of both cultivars were *F. avenaceum*, *F. culmorum*, *F. poae*, *F. sporotrichioides*, *F. tricinctum*, and *F. equiseti*. Delayed harvesting led to a significant increase in infection by *F. poae*, *F. sporotrichioides*, *F. equiseti*, *F. culmorum*, and *F. tricinctum* (*p* < 0.05) (Figure 3). Comparing the first and third harvests in 2020 and 2021, *F. poae* and *F. sporotrichioides* infections increased by an average of 24% and 16.3%, respectively. In 2021, infections by *F. equiseti*, *F. culmorum*, and *F. tricinctum* increased by 12.4%, 9.9%, and 20.9%, respectively. In contrast, when rainy conditions prevailed during the growing season but were followed by dry and warm conditions at harvest (in 2022), *Fusarium* spp. infection levels remained low. However, an increase in *F. avenaceum* (by 5.1%), *F. poae* (by 9.8%), and *F. sporotrichioides* (by 4%) infections was still observed in the later harvests (*p* < 0.05).

The nutritional composition of spring barley grain at different harvest times is shown in Table 2. Significant differences in nutritive value composition between the ‘Luokė’ and ‘Laureate’ varieties were observed in 2021 and 2022. The grain of ‘Luokė’ had a CP content that was 0.6–0.8% higher in DM (*p* ≤ 0.05) and a CF content that was 1.3% higher in DM (*p* ≤ 0.001). However, the grain of ‘Luokė’ had a starch content that was 5.1% lower in DM compared to the grain of ‘Laureate’ (*p* ≤ 0.001).

In 2020, when precipitation and temperature were slightly above the long-term average during the barley flowering period and similar to the long-term average during the harvest period, the DM content of the ‘Laureate’ grain remained stable across the harvests. However, the CF and CA contents increased significantly by 39% (*p* ≤ 0.05) and 32% (*p* ≤ 0.001), respectively, compared to the first harvest. Conversely, there was a notable decrease in starch content with delayed harvesting, particularly at the third harvest, where the starch content decreased by an average of 8% compared to the first two harvests. For ‘Luokė’, the CA content also increased significantly at the second and third harvests, averaging 23% higher than at the first harvest (*p* ≤ 0.001). However, other nutritive value indicators showed different trends. The DM content decreased significantly at both the second and third harvests (*p* ≤ 0.001). Notably, the starch content increased significantly at the third harvest, with an average increase of 8% compared to the first two harvests (*p* ≤ 0.001).

In 2021, the meteorological conditions featured dry and warm weather during the flowering period and rainy and cool conditions during the harvest period. Under these conditions, delaying the harvest led to average increases in DM (*p* ≤ 0.01), crude fat (*p* ≤ 0.01), and CA (*p* ≤ 0.05) contents by 2%, 51%, and 19%, respectively. However, the CP content decreased significantly by 7% on average (*p* ≤ 0.05) in both varieties in 2021. Similar to 2020, the starch content decreased significantly by 3% in the ‘Laureate’ grain, while in the ‘Luokė’ grain, it increased significantly by 7% (*p* ≤ 0.01).

In 2022, with rainy and wet conditions during the barley flowering period and dry and warm conditions during the harvest period, the results indicated a slight increase in DM content (*p* ≤ 0.05) in the grains of both varieties by an average of 1%. For ‘Laureate’, the CF (*p* ≤ 0.01) and CA (*p* ≤ 0.05) contents increased significantly by 40% and 8%, respectively. In contrast, the CF content in the ‘Luokė’ grain did not change significantly, and the CA content decreased by 12% (*p* ≤ 0.05). The starch content decreased by 4% in the ‘Laureate’ grain (*p* ≤ 0.05), while it remained stable in ‘Luokė’.

Only slight differences in mineral content were observed between varieties (Table 3). In 2020, the grain of the ‘Luokė’ exhibited a higher Fe content of 22 mg kg^−1^ (*p* ≤ 0.01). In 2021, no differences in mineral content were observed between the varieties. However, in 2022, the P content of the grain of the ‘Luokė’ variety was 245 mg kg^−1^ higher than that of the ‘Laureate’ variety (*p* ≤ 0.01).

In 2020, a significant decrease of 265 mg kg^−1^ in Mg content was observed at the second harvest (*p* ≤ 0.05) of the ‘Luokė’ variety. Additionally, there was a decrease of 690 to 815 mg kg^−1^ in Ca content at the second and third harvests, respectively (*p* ≤ 0.05). However, the Zn content increased significantly by 16 mg kg^−1^ at the third harvest (*p* ≤ 0.01). In the ‘Laureate’ variety, a significant increase in P content was noted, ranging from 720 to 1260 mg kg^−1^ at the second and third harvests (*p* ≤ 0.01).

In 2021, the ‘Luokė’ variety exhibited a decrease in Zn content at both the second and third harvests, with reductions from 23 to 25 mg kg^−1^ (*p* ≤ 0.05). The same trend was determined in the ‘Laureate’ variety; there was a significant decrease in Zn content, ranging from 19 to 27 mg kg^−1^ (*p* ≤ 0.001). However, Fe content increased by an average of 17.5 mg kg^−1^ in the second and third harvests (*p* ≤ 0.05). In 2022, the Luokė variety continued to show a decrease in Zn content with delayed harvests, with reductions ranging from 8 to 11 mg kg^−1^ (*p* ≤ 0.05). Additionally, a decrease of 40 mg kg^−1^ in Fe content was observed in the third harvest (*p* ≤ 0.01). For the ‘Laureate’ variety, there was a significant decrease in Mg content by 151 mg kg^−1^ and Ca content by 630 mg kg^−1^ at the third harvest (*p* ≤ 0.001). Additionally, a significant reduction by 19 and 52 mg kg^−1^ in Fe content was observed at the second (*p* ≤ 0.01) and third harvests (*p* ≤ 0.001).

Regarding mycotoxin contamination, differences between the varieties’ significant variation was observed over the three-year study period (Table 4). In 2020, ‘Laureate’ exhibited a 10 µg kg^−1^ higher concentration of MON compared to ‘Luokė’. Conversely, the concentrations of ZEA, NIV, and ENN A were 75, 14, and 13 µg kg^−1^ lower, respectively, in ‘Laureate’. In 2021, the only notable difference was in the concentration of ENN B1, which was reduced by half in ‘Laureate’, measuring at 23 µg kg^−1^. The 2022 survey revealed higher concentrations of ENNs in the ‘Laureate’ grain. Specifically, the concentrations of ENN B, ENN B1, and ENN A1 were significantly higher by 128, 87, and 11 µg kg^−1^, respectively.

In the 2020 harvest, DON was exclusively detected in the latest harvested grain of ‘Laureate’. T-2 toxin was also found in a limited number of samples that year, with the highest concentrations observed in ‘Luokė’, particularly during the first and second harvests. Mycotoxin HT-2 was also consistently present across all harvests in 2020, with some samples showing elevated levels; however, delayed harvesting did not yield significant differences. The 2021 HT-2 concentrations were slightly above the LOQ and did not exhibit significant differences across various harvest times. The highest concentrations of ZEA were observed for that year for both barley varieties. Significant changes in ZEA concentration were noted only in ‘Luokė’, where the latest harvest showed levels averaging 288 µg kg^−1^ higher than the first two harvests. AFLs (B1 + B2 + G1 + G2) above the LOQ were found in both 2020 and 2021. In these years, concentrations below the LOQ were observed in the first harvests of both varieties. However, higher concentrations were detected in ‘Laureate’ grain during second and third harvests. For ‘Luokė’, concentrations above the LOQ were only detected in the third harvest, notably increasing to 4.7 µg kg^−1^ in 2020 and 2.4 µg kg^−1^ in 2021 (*p* ≤ 0.05). In contrast, no significant differences were observed in the concentrations of MON, NIV, or ENNs A, A1, B, or B1 between the barley harvests of 2020 and 2021, indicating that delayed harvesting did not significantly influence these mycotoxins. However, in 2022, there was a significant increase in the concentrations of NIV and ENNs in the third harvest. Specifically, in the ‘Laureate’ grain, the concentrations of NIV and ENNs A, A1, B, and B1 increased by 205, 62, 23, 120, and 132 µg kg^−1^, respectively, from the first to the third harvest (*p* ≤ 0.05). Similarly, for ‘Luokė’, ENN concentrations in the third harvest increased by 46, 12, 72, and 100 µg kg^−1^ for ENNs A, A1, B, and B1, respectively, compared to the first two harvests (*p* ≤ 0.05).

**Figure 4:**
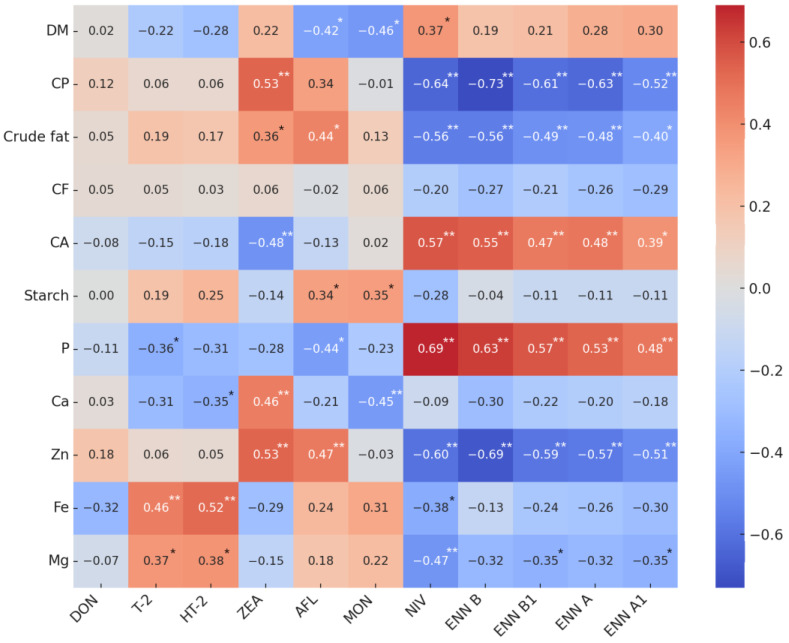
Correlation heatmap between barley grain nutrition value indicators and mycotoxins. Note. Positive correlations are shown in red, indicating positive relationships, while negative correlations are shown in blue, indicating negative relationships. The intensity of the color represents the strength of the correlation. * *p* ≤ 0.05, ** *p* ≤ 0.01.

The heatmap in Figure 4 illustrates the correlations between quality indicators and mycotoxin contamination in barley. There were no consistent trends where all mycotoxins exhibited a uniform effect on specific quality indicators. However, distinct groupings were discerned, particularly between T-2 and HT-2, and ENNs and NIV, suggesting similar correlations, likely due to their co-occurrence. While many correlations were not statistically significant, several notable associations were observed. Both T-2 and HT-2 showed positive correlations with Fe (r = 0.46 and r = 0.52, respectively, *p* ≤ 0.01). ZEA exhibited positive correlations with CP (r = 0.53, *p* ≤ 0.01), Ca (r = 0.46, *p* ≤ 0.01), and Zn (r = 0.53, *p* ≤ 0.01), along with a negative correlation with CA (r = −0.48, *p* ≤ 0.01). AFL was positively correlated with crude fat (r = 0.44, *p* ≤ 0.01) and Zn (r = 0.47, *p* ≤ 0.01), but showed a negative correlation with P (r = −0.44, *p* ≤ 0.01). MON was negatively correlated with DM (r = 0.46, *p* ≤ 0.01) and Ca (r = −0.45, *p* ≤ 0.01). Enniatin mycotoxins (B, B1, A, A1) and NIV consistently showed negative correlations with CP (r = −0.63, *p* ≤ 0.01), crude fat (r = −0.50, *p* ≤ 0.01), and Zn (r = −0.59, *p* ≤ 0.01), while displaying positive correlations with CA (r = 0.49, *p* ≤ 0.01) and P (r = 0.58, *p* ≤ 0.01).

## 4. Discussion

Each year, delayed harvesting resulted in an increase in infection rates of key *Fusarium* species, such as *F. culmorum*, *F. poae*, *F. sporotrichioides*, *F. tricinctum*, and *F. equiseti*. This increase was notably higher during harvests that followed rainy periods. This is consistent with previous studies, which also identified these species as some of the most frequently occurring in barley crops under favorable weather conditions for fungal growth [36,37]. Furthermore, research conducted in Canada similarly observed that delayed harvests led to a significant increase in *Fusarium* spp. infection in barley, with infection levels rising notably due to prolonged exposure to rainy conditions [38].

The barley variety ‘Luokė’ exhibited higher CP and CF, but lower starch content compared to ‘Laureate’. This variation aligns with the findings by Deme et al. that indicate that malt barley grain typically has higher starch and lower protein levels [6]. ‘Laureate’, selected for this study, is classified as a malting grain, whereas ‘Luokė’ is predominantly used for feed. During the 2021 study year, characterized by drier and warmer weather, the lowest CA content and the highest CP content were recorded. Conversely, in the wetter 2022 season, the lowest CP and crude fat contents were observed, along with the highest starch content. This observation is supported by research from Izydorczyk et al. [39] and Ben Mariem et al. [40], who found that drought conditions and high temperatures can significantly elevate the protein and mineral content in barley and other cereals, while reducing starch content.

Additionally, several trends were noted at late harvest, including increased DM, crude fat, and CA content, while CP content generally remained stable or decreased significantly. Mineral content exhibited minimal individual variation between ‘Luokė’ and ‘Laureate’. However, interannual differences were observed in mineral content: Mg and Fe levels were higher in 2020, a year without extreme droughts or heavy rainfalls, with harvest conditions similar to the long-term average. In contrast, Ca and Zn levels were elevated in the drier year of 2021, which had a rainy harvest period, while P content was higher in the wetter year of 2022, which saw a drier harvest period. With delayed harvesting, the levels of Mg, Ca, and P in both varieties remained relatively stable, whereas Zn and Fe levels showed a declining trend, particularly in the rainy year of 2022. Although changes in quality parameters and mineral content due to delayed harvesting were not extensively studied, a previous study in Lithuania on wheat grain indicated that delayed harvest tends to increase specific metrics such as DM, hectoliter weight, and CP, while decreasing mineral content, including Ca and P [20].

Throughout the study, barley grain exhibited a diverse spectrum of mycotoxins, with distinct variations observed each year. Interestingly, when slightly warmer and wetter weather conditions occurred during flowering, and harvest conditions were similar to the long-term average, the concentrations of HT-2 toxin were significantly higher. In contrast, elevated ZEA concentrations were detected during dry and warm weather conditions during the growing season, followed by rainy and cool weather at harvest. Additionally, significantly higher concentrations of NIV and ENN B, B1, A, and A1 were associated with rainy weather during the growing season and dry, warm conditions at harvest. These findings are consistent with previous research. Studies from Germany and France have reported higher levels of *F. avenaceum* contamination and, correspondingly, higher ENN contamination in barley and oat grains following prolonged rainy periods [24,41]. Similarly, Estonian researchers have observed that higher concentrations of T-2 and HT-2 were detected during typical weather conditions, without extreme droughts or heavy rainfall. Specifically, during the growing season characterized by dry and warm weather but with rainy and cool conditions at harvest, only trace amounts of T-2 and HT-2 were found in barley grain, whereas ZEA concentrations increased significantly [42,43].

During our study, higher concentrations of various mycotoxins were observed in different grain varieties, though no consistent trend indicated a predisposition for higher contamination in any specific variety. The concentrations of DON, T-2 toxin, HT-2 toxin, and ZEA did not significantly change with delayed harvesting. Although *F. culmorum* was detected—particularly at the latest harvest in 2021, where up to 10% of grains were infected—DON concentrations remained below the LOQ throughout the study. This suggests that despite the presence of a known DON producer, environmental conditions may have contributed to the absence of significant DON accumulation, as previous studies have shown that weather conditions can strongly influence the correlation between *F. culmorum* infection and DON production [44]. However, in years when AFLs (B1 + B2 + G1 + G2), NIV, and ENNs (B, B1, A, A1) were detected, these mycotoxins tended to increase in concentration. Swedish researchers also studied the changes in concentrations of DON, ZEA, T-2, and HT-2 toxins in barley grain with a one-week delay in harvest and found no significant changes. However, they observed an increase in the concentrations of DON and ZEA in maize and wheat grain under similar conditions [44].

Our study revealed that mycotoxin contamination in barley shows variable correlations with quality indicators. The strongest correlations were observed with the less-studied mycotoxins, such as NIV and ENNs. Although scientific studies on the subject are limited, the most commonly observed negative effects of mycotoxins include the negative correlations of T-2 toxin with CP, Ca, and P [45]. In studies of other grain types, researchers recorded negative correlations between various AFLs and crude fat, along with other nutritive value indicators [46,47]. Additionally, researchers in the United States of America found that corn samples contaminated with DON exhibited significantly higher starch levels and lower protein levels, while ZEA-contaminated samples showed significantly lower starch content [47]. In Lithuania, research investigating the relationships between mycotoxins and nutritive value indicators in wheat also identified isolated negative correlations, though these lacked consistent patterns [48].

This study employed morphological methods to identify *Fusarium* species, which, while effective, may lack the precision of molecular techniques like PCR that can provide more accurate strain identification. Additionally, correlations between barley grain nutritive value indicators and mycotoxin concentrations were influenced by instances where mycotoxin levels did not reach the LOQ, and LOQ/2 values were used for calculations. This standard approach, though necessary for handling low-concentration data, may have introduced some uncertainty into the observed correlations between barley grain nutritive value indicators and mycotoxin levels.

## 5. Conclusions

Harvesting under rainier weather conditions significantly increased the infection rates of key *Fusarium* species, such as *F. culmorum*, *F. poae*, *F. sporotrichioides*, *F. tricinctum*, and *F. equiseti*, and delaying the harvest also had a significant effect on these infection rates. Barley intended for malting generally exhibited higher starch and lower protein content, a finding reinforced by comparing malting barley with feed barley in this study. The impact of delayed harvest on nutritive value indicators was also significant, resulting in increased levels of dry matter, crude fat, and crude ash contents, and decreased levels of crude protein, zinc, and iron. Mycotoxin analysis revealed distinct patterns under specific weather conditions. HT-2 toxin levels were higher under slightly warmer and wetter conditions during flowering, with harvest conditions similar to the long-term average. Zearalenone increased following dry and warm growing seasons with subsequent rainy harvests. Nivalenol and enniatins (B, B1, A, A1) increased during rainy growing seasons and dry, warm harvests. No consistent trend indicated a predisposition for higher contamination in any specific barley variety. The strongest correlations between mycotoxins and nutritive value indicators were observed with the less-studied mycotoxins, such as nivalenol and enniatins (B, B1, A, A1), showing negative correlations with crude protein, crude fat, and zinc, and positive correlations with crude ash and phosphorus.

## Figures and Tables

**Table 1:** Spring barley harvest times in 2020–2022.

Years	1st Harvest	2nd Harvest	3rd Harvest
2020	15 August 2020	25 August 2020	5 September 2020
	Hard maturity *	Hard maturity +10 days	Hard maturity +21 days
2021	1 August 2021	11 August 2021	19 August 2021
	Hard maturity *	Hard maturity +10 days	Hard maturity +18 days
2022	12 August 2022	21 August 2022	3 August 2022
	Hard maturity *	Hard maturity +9 days	Hard maturity +22 days

* BBCH 89—the grain is hard and difficult to press with a fingernail.

**Table 2:** Changes in dry matter, starch, crude protein, crude fat, crude ash, and crude fiber contents in barley grains of different varieties at different harvest times from 2020 to 2022.

Variety	Harvest	Dry Matter (%)	Starch	Crude Protein	Crude Fat	Crude Ash	Crude Fiber
			% in Dry Matter				
2020							
‘Luokė’	1st	88.4 ^a^ ± 0.3	60.4 ^b^ ± 0.4	11.8 ^a^ ± 0.6	2.6 ^a^ ± 0.1	2.0 ^b^ ± 0.01	6.3 ^ab^ ± 0.7
	2nd	87.2 ^b^ ± 0.2	58.7 ^c^ ± 0.6	11.6 ^a^ ± 0.7	2.5 ^a^ ± 0.1	2.4 ^a^ ± 0.1	6.8 ^a^ ± 0.4
	3rd	86.7 ^c^ ± 0.4	64.1 ^a^ ± 0.1	11.5 ^a^ ± 0.5	2.4 ^b^ ± 0.1	2.5 ^a^ ± 0.1	5.6 ^b^ ± 0.2
	Mean	87.4 ^A^ ± 0.8	61.1 ^A^ ± 2.4	11.6 ^A^ ± 0.6	2.5 ^A^ ± 0.1	2.3 ^A^ ± 0.2	6.2 ^A^ ± 0.7
‘Laureate’	1st	87.1 ^a^ ± 0.2	64.2 ^a^ ± 0.2	11.9 ^a^ ± 0.3	2.4 ^b^ ± 0.1	1.9 ^c^ ± 0.01	4.9 ^c^ ± 0.4
	2nd	86.6 ^b^ ± 0.2	64.1 ^a^ ± 0.3	11.7 ^a^ ± 0.6	2.5 ^a^ ± 0.1	2.3 ^b^ ± 0.1	6.3 ^b^ ± 0.5
	3rd	87.3 ^a^ ± 0.4	58.9 ^b^ ± 0.5	11.5 ^a^ ± 0.6	2.5 ^ab^ ± 0.1	2.7 ^a^ ± 0.1	7.3 ^a^ ± 0.6
	Mean	87.0 ^A^ ± 0.4	62.4 ^A^ ± 2.6	11.7 ^A^ ± 0.5	2.4 ^A^ ± 0.1	2.3 ^A^ ± 0.4	6.2 ^A^ ± 1.1
2021							
‘Luokė’	1st	87.6 ^b^ ± 0.4	57.0 ^b^ ± 1.1	13.9 ^a^ ± 0.6	2.0 ^c^ ± 0.1	1.7 ^b^ ± 0.01	6.9 ^a^ ± 0.2
	2nd	88.7 ^a^ ± 0.2	55.6 ^b^ ± 1.4	13.7 ^a^ ± 0.4	3.0 ^b^ ± 0.3	2.0 ^a^ ± 0.1	6.9 ^a^ ± 0.6
	3rd	88.8 ^a^ ± 0.1	60.4 ^a^ ± 1.1	12.6 ^b^ ± 0.1	3.7 ^a^ ± 0.1	2.1 ^a^ ± 0.1	5.9 ^b^ ± 0.1
	Mean	88.3 ^A^ ± 0.6	57.1 ^B^ ± 2.0	13.5 ^A^ ± 0.7	2.8 ^A^ ± 0.7	1.9 ^A^ ± 0.2	6.7 ^A^ ± 0.6
‘Laureate’	1st	87.4 ^b^ ± 0.1	62.2 ^a^ ± 1.1	12.9 ^a^ ± 0.1	2.3 ^b^ ± 0.2	1.7 ^b^ ± 0.01	5.2 ^a^ ± 0.4
	2nd	88.8 ^a^ ± 0.3	60.4 ^b^ ± 0.9	13.2 ^a^ ± 0.3	3.2 ^a^ ± 0.2	1.9 ^a^ ± 0.1	5.5 ^a^ ± 0.4
	3rd	89.1 ^a^ ± 0.1	60.4 ^b^ ± 0.1	12.4 ^b^ ± 0.1	3.1 ^a^ ± 0.1	2.1 ^a^ ± 0.1	5.7 ^a^ ± 0.1
	Mean	88.3 ^A^ ± 0.8	61.1 ^A^ ± 1.0	12.9 ^B^ ± 0.4	2.8 ^A^ ± 0.5	1.9 ^A^ ± 0.2	5.4 ^B^ ± 0.4
2022							
‘Luokė’	1st	88.1 ^b^ ± 0.4	55.8 ^a^ ± 1.2	10.9 ^a^ ± 0.2	1.6 ^a^ ± 0.1	2.9 ^a^ ± 0.2	6.9 ^a^ ± 0.6
	2nd	88.9 ^a^ ± 0.4	55.5 ^a^ ± 1.7	11.0 ^a^ ± 0.2	1.8 ^a^ ± 0.3	2.6 ^b^ ± 0.1	6.6 ^a^ ± 0.8
	3rd	88.7 ^a^ ± 0.1	55.3 ^a^ ± 0.9	11.3 ^a^ ± 0.2	1.8 ^a^ ± 0.3	2.5 ^b^ ± 0.3	6.4 ^a^ ± 0.5
	Mean	88.6 ^A^ ± 0.5	55.5 ^B^ ± 1.2	11.0 ^A^ ± 0.4	1.8 ^A^ ± 0.2	2.7 ^A^ ± 0.3	6.6 ^A^ ± 0.6
‘Laureate’	1st	88.1 ^b^ ± 0.3	62.1 ^a^ ± 1.2	10.0 ^a^ ± 0.5	1.5 ^b^ ± 0.2	2.4 ^b^ ± 0.1	5.9 ^a^ ± 1.0
	2nd	88.6 ^a^ ± 0.2	59.9 ^b^ ± 1.7	10.3 ^a^ ± 0.3	1.8 ^ab^ ± 0.3	2.4 ^ab^ ± 0.2	5.1 ^a^ ± 0.2
	3rd	88.6 ^a^ ± 0.1	59.7 ^b^ ± 0.9	10.4 ^a^ ± 0.3	2.1 ^a^ ± 0.3	2.6 ^a^ ± 0.2	4.9 ^a^ ± 0.2
	Mean	88.5 ^A^ ± 0.3	60.6 ^A^ ± 1.6	10.2 ^B^ ± 0.4	1.8 ^A^ ± 0.4	2.5 ^A^ ± 0.2	5.3 ^B^ ± 0.9

Note. Different lowercase letters indicate statistically significant distinctions in nutritive value composition among harvests within the same year and variety of grain. Different uppercase letters denote statistically significant variations in nutritive value composition observed among varieties within the same study year.

**Table 3:** Variations in magnesium, calcium, phosphorus, zinc, and iron levels at different harvest times in barley grain varieties from 2020 to 2022.

Variety	Harvest	Magnesium	Calcium	Phosphorus	Iron	Zinc
		(mg kg^−1^)				
2020						
‘Luokė’	1st	2303 ^a^ ± 205	3090 ^a^ ± 247	3125 ^a^ ± 261	124 ^a^ ± 14	28 ^b^ ± 1
	2nd	2038 ^b^ ± 85	2400 ^b^ ± 179	3538 ^a^ ± 249	125 ^a^ ± 5	28 ^b^ ± 4
	3rd	2100 ^ab^ ± 82	2275 ^b^ ± 236	3450 ^a^ ± 173	142 ^a^ ± 11	44 ^a^ ± 7
	Mean	2147 ^A^ ± 171	2588 ^A^ ± 297	3371 ^A^ ± 257	131 ^A^ ± 13	33 ^A^ ± 9
‘Laureate’	1st	2100 ^a^ ± 180	2595 ^a^ ± 207	2753 ^b^ ± 296	124 ^a^ ± 6	27 ^a^ ± 1
	2nd	2043 ^a^ ± 131	2345 ^a^ ± 115	4015 ^a^ ± 286	110 ^a^ ± 23	29 ^a^ ± 5
	3rd	2075 ^a^ ± 171	2325 ^a^ ± 279	3475 ^a^ ± 386	95 ^a^ ± 23	32 ^a^ ± 3
	Mean	2073 ^A^ ± 149	2422 ^A^ ± 291	3414 ^A^ ± 331	109 ^B^ ± 21	29 ^A^ ± 4
2021						
‘Luokė’	1st	1925 ^a^ ± 50	3400 ^a^ ± 183	3350 ^a^ ± 182	71 ^a^ ± 16	66 ^a^ ± 5
	2nd	2000 ^a^ ± 62	3650 ^a^ ± 58	3200 ^a^ ± 332	83 ^a^ ± 15	43 ^b^ ± 13
	3rd	1950 ^a^ ± 71	3500 ^a^ ± 141	2950 ^a^ ± 140	97 ^a^ ± 2	41 ^b^ ± 1
	Mean	1960 ^A^ ± 51	3520 ^A^ ± 169	3210 ^A^ ± 300	81 ^A^ ± 17	52 ^A^ ± 15
‘Laureate’	1st	2025 ^a^ ± 50	3750 ^a^ ± 238	3200 ^a^ ± 264	73 ^b^ ± 7	61 ^a^ ± 4
	2nd	1925 ^a^ ± 50	3550 ^a^ ± 100	2800 ^b^ ± 141	72 ^b^ ± 7	34 ^c^ ± 2
	3rd	2000 ^a^ ± 141	3900 ^a^ ± 141	3300 ^a^ ± 178	90 ^a^ ± 2	42 ^b^ ± 1
	Mean	1980 ^A^ ± 79	3700 ^A^ ± 211	3060 ^A^ ± 259	76 ^A^ ± 9	46 ^A^ ± 13
2022						
‘Luokė’	1st	1915 ^a^ ± 47	3025 ^a^ ± 186	4695 ^a^ ± 115	90 ^a^ ± 8	24 ^a^ ± 6
	2nd	1965 ^a^ ± 66	3255 ^a^ ± 239	4885 ^a^ ± 139	86 ^a^ ± 16	13 ^b^ ± 1
	3rd	1903 ^a^ ± 46	3065 ^a^ ± 60	4778 ^a^ ± 160	48 ^b^ ± 16	16 ^b^ ± 2
	Mean	1928 ^A^ ± 76	3115 ^A^ ± 230	4786 ^A^ ± 150	75 ^A^ ± 23	17 ^A^ ± 6
‘Laureate’	1st	1903 ^a^ ± 59	3178 ^a^ ± 110	4435 ^a^ ± 204	106 ^a^ ± 15	15 ^a^ ± 5
	2nd	1925 ^a^ ± 19	3178 ^a^ ± 172	4673 ^a^ ± 95	87 ^b^ ± 13	13 ^a^ ± 2
	3rd	1763 ^b^ ± 15	2548 ^b^ ± 78	4515 ^a^ ± 149	55 ^b^ ± 7	15 ^a^ ± 1
	Mean	1863 ^A^ ± 82	2968 ^A^ ± 230	4541 ^B^ ± 175	83 ^A^ ± 25	14 ^A^ ± 3

Note. Different lowercase letters indicate statistically significant distinctions in mineral content observed among harvests within the same year and variety of grain. Different uppercase letters denote statistically significant variations in mineral content observed among varieties within the same study year.

**Table 4:** Variations in concentrations (µg kg^−1^) of deoxynivalenol, T-2, HT-2, zearalenone, aflatoxins, moniliformin, nivalenol, and enniatins B, B1, A, and A1 at delayed harvest times in barley grain of ‘Luokė’ and ‘Laureate’ varieties from 2020 to 2022.

Mycotoxin	‘Luokė’				‘Laureate’			
	1st Harvest	2nd Harvest	3rd Harvest	Mean	1st Harvest	2nd Harvest	3rd Harvest	Mean
2020								
Deoxynivalenol	<LOQ	<LOQ	<LOQ	<LOQ	<LOQ	<LOQ	<LOQ	<LOQ
T-2	53 ^a^ ± 17	36 ^a^ ± 9	<LOQ	35 ± 8	27 ± 7	<LOQ	<LOQ	<LOQ
HT-2	159 ^a^ ± 96	126 ^a^ ± 42	50 ^a^ ± 23	112 ^A^ ± 21	85 ^a^ ± 33	42 ^a^ ± 15	<LOQ	47 ^B^ ± 10
Zearalenone	<LOQ	<LOQ	<LOQ	<LOQ	<LOQ	<LOQ	<LOQ	<LOQ
Aflatoxins B1 + B2 + G1 + G2	<LOQ	<LOQ	4.7 ± 0.2	2.0 ^A^ ± 0.6	<LOQ	1.8 ^b^ ± 0.1	2.5 ^a^ ± 0.3	1.8 ^A^ ± 0.3
Moniliformin	3 ^a^ ± 2	3 ^a^ ± 2	<LOQ	2 ^B^ ± 1	11 ^a^ ± 3	14 ^a^ ± 6	11 ^a^ ± 3	12 ^A^ ± 2
Nivalenol	40 ^a^ ± 5	51 ^a^ ± 9	39 ^a^ ± 5	43 ^A^ ± 4	26 ^a^ ± 8	31 ^a^ ± 7	30 ^a^ ± 9	29 ^B^ ± 4
Enniatin B	101 ^a^ ± 19	114 ^a^ ± 16	103 ^a^ ± 13	106 ^A^ ± 9	83 ^a^ ± 15	84 ^a^ ± 16	74 ^a^ ± 18	81 ^A^ ± 9
Enniatin B1	89 ^a^ ± 23	88 ^a^ ± 25	85 ^a^ ± 21	87 ^A^ ± 12	90 ^a^ ± 21	88 ^a^ ± 15	89 ^a^ ± 19	89 ^A^ ± 10
Enniatin A	34 ^a^ ± 9	25 ^a^ ± 9	29 ^a^ ± 11	29 ^A^ ± 5	15 ^a^ ± 2	16 ^a^ ± 2	16 ^a^ ± 4	16 ^B^ ± 2
Enniatin A1	7 ^a^ ± 1	<LOQ	<LOQ	<LOQ	<LOQ	<LOQ	<LOQ	<LOQ
2021								
Deoxynivalenol	<LOQ	<LOQ	<LOQ	<LOQ	<LOQ	<LOQ	<LOQ	<LOQ
T-2	<LOQ	<LOQ	<LOQ	<LOQ	<LOQ	<LOQ	<LOQ	<LOQ
HT-2	<LOQ	<LOQ	<LOQ	<LOQ	<LOQ	<LOQ	<LOQ	<LOQ
Zearalenone	469 ^b^ ± 103	393 ^b^ ± 239	719 ^a^ ± 250	488 ^A^ ± 110	362 ^a^ ± 187	427 ^a^ ± 141	511 ^a^ ± 52	433 ^A^ ± 138
Aflatoxins B1 + B2 + G1 + G2	1.7 ^b^ ± 0.1	1.7 ^b^ ± 0.2	2.4 ^a^ ± 0.1	1.8 ^A^ ± 0.1	<LOQ	2.2 ^a^ ± 0.3	2.1 ^a^ ± 0.1	1.8 ^A^ ± 0.2
Moniliformin	<LOQ	<LOQ	<LOQ	<LOQ	<LOQ	<LOQ	<LOQ	<LOQ
Nivalenol	16 ^a^ ± 7	23 ^a^ ± 9	21 ^a^ ± 11	20 ^A^ ± 5	<LOQ	17 ^a^ ± 8	12 ^a^ ± 8	13 ^A^ ± 4
Enniatin B	<LOQ	<LOQ	<LOQ	<LOQ	<LOQ	<LOQ	<LOQ	<LOQ
Enniatin B1	57 ^a^ ± 16	59 ^a^ ± 15	51 ^a^ ± 17	57 ^A^ ± 8	21 ^a^ ± 5	26 ^a^ ± 9	21 ^a^ ± 11	23 ^B^ ± 4
Enniatin A	<LOQ	<LOQ	<LOQ	<LOQ	<LOQ	<LOQ	<LOQ	<LOQ
Enniatin A1	<LOQ	<LOQ	<LOQ	<LOQ	<LOQ	<LOQ	<LOQ	<LOQ
2022								
Deoxynivalenol	<LOQ	<LOQ	<LOQ	<LOQ	<LOQ	<LOQ	<LOQ	<LOQ
T-2	<LOQ	<LOQ	<LOQ	<LOQ	<LOQ	<LOQ	<LOQ	<LOQ
HT-2	<LOQ	<LOQ	<LOQ	<LOQ	<LOQ	<LOQ	<LOQ	<LOQ
Zearalenone	<LOQ	52 ^a^ ± 49	71 ^a^ ± 38	49 ^A^ ± 15	47 ^a^ ± 11	36 ^a^ ± 7	<LOQ	35 ^A^ ± 6
Aflatoxins B1 + B2 + G1 + G2	<LOQ	<LOQ	<LOQ	<LOQ	<LOQ	<LOQ	<LOQ	<LOQ
Moniliformin	<LOQ	<LOQ	<LOQ	<LOQ	<LOQ	<LOQ	<LOQ	<LOQ
Nivalenol	232 ^a^ ± 70	187 ^a^ ± 14	231 ^a^ ± 33	217 ^A^ ± 25	196 ^b^ ± 42	225 ^b^ ± 31	401 ^a^ ± 78	274 ^A^ ± 39
Enniatin B	155 ^b^ ± 24	119 ^b^ ± 32	209 ^a^ ± 30	161 ^B^ ± 20	219 ^b^ ± 24	299 ^ab^ ± 76	339 ^a^ ± 50	289 ^A^ ± 32
Enniatin B1	137 ^b^ ± 29	93 ^b^ ± 10	215 ^a^ ± 35	148 ^B^ ± 21	171 ^b^ ± 18	232 ^ab^ ± 49	303 ^a^ ± 58	235 ^A^ ± 29
Enniatin A	49 ^b^ ± 13	26 ^b^ ± 4	83 ^a^ ± 13	53 ^A^ ± 9	56 ^b^ ± 7	77 ^ab^ ± 18	118 ^a^ ± 29	84 ^A^ ± 13
Enniatin A1	8 ^b^ ± 5	<LOQ	20 ^a^ ± 3	10 ^A^ ± 3	11 ^b^ ± 4	18 ^ab^ ± 4	34 ^a^ ± 7	21 ^A^ ± 4

Note. Different lowercase letters indicate statistically significant distinctions in mineral content observed among harvests within the same year and variety of grain. Different uppercase letters denote statistically significant variations in mineral content observed among varieties within the same study year. <LOQ—below limit of quantification.

## Data Availability

The original contributions presented in the study are included in the article, further inquiries can be directed to the corresponding author.
